# Teach Our Children: Stroke Education for Indigenous Children, First Nations, Ontario, Canada, 2009–2012

**DOI:** 10.5888/pcd14.160506

**Published:** 2017-08-17

**Authors:** Mary Ellen Hill, Pauline Bodnar, Robert Fenton, Brenda Mason, Grace Bandoh

**Affiliations:** 1 Centre for Rural and Northern Health Research, Lakehead University, Thunder Bay, Ontario, Canada; 2 Northwestern Ontario Regional Stroke Network, Thunder Bay, Ontario, Canada; 3 Animakee Wa Zhing First Nation; Aboriginal Advisory Committee, Northwestern Ontario Regional Stroke Network; Diabetes Educator, Ontario Native Women’s Association; Thunder Bay, Ontario, Canada; 4 Sandy Lake First Nation; Aboriginal Advisory Committee, Northwestern Ontario Regional Stroke Network, Thunder Bay, Ontario, Canada

## Abstract

**Background:**

Because of the heightened risk for stroke among indigenous people, we conducted this multiyear community case study from 2009 through 2012 to address stroke education needs among children aged 11 to 13 years residing in northern urban, rural, and remote First Nations in Ontario, Canada. The goal was to determine what young people understand about stroke and to develop an age-appropriate and culturally appropriate educational product.

**Community Context:**

This project responded to First Nations requests that we educate their young people about the signs and symptoms of stroke and the need for early response. Ten First Nations and 4 indigenous health organizations took part; 7 contributed to the educational product.

**Methods:**

This study was developed under the guidance of the Northwestern Ontario Regional Stroke Network Aboriginal Advisory Committee. It employed indigenous researchers and facilitators to ensure that methods used (questions assessing children’s knowledge of stroke and their ideas on how best to deliver messages) reflected the cultural values of participating study sites.

**Outcome:**

Indigenous children had limited knowledge about stroke and its signs, symptoms, and consequences; children in remote communities were better informed than those in other locations. Educators agreed that a DVD was the most effective way to deliver stroke information to children in this age group. The principal outcome from this 3-year community engagement was an 11.5-minute DVD titled Act F-A-S-T 1-2-3!. Follow-up indicated that the educational tool continued to be used to educate indigenous children and adults about stroke signs and symptoms, the need for early response, and risk reduction.

**Interpretation:**

Although indigenous communities are each unique in their culture and traditions, all have a strong commitment to improving health and are generous in their support for research that addresses their needs. Our study provides examples of the engagement and participatory research strategies that were effective, the practical supports required, limitations to the study, and how barriers to stroke education can be overcome.

## Background

Because indigenous people worldwide are at heightened risk of death from cardiovascular diseases, culturally appropriate education can strengthen preventive care (1). Evidence from the United States (2) and Canada (3) confirms that indigenous people have up to twice the risk of stroke, compared with nonindigenous people; because of a higher prevalence of diabetes, they also have a higher risk of experiencing stroke at younger ages (4,5). Although culturally tailored cardiovascular disease prevention programs have been developed for indigenous communities, such as the Honoring the Gift of Heart Health Curriculum (6), Montana Cardiovascular Health Program (7), or In Our Voice Curriculum for American Indian Students (8), evaluations indicate that such programs often do not produce expected improvements in stroke awareness, unless culturally specific and community-relevant content is offered.

Teach Our Children is a participatory community-based research and education project (9) that arose from a request by elders who took part in a 2007 through 2009 health promotion initiative of the Northwestern Ontario Regional Stroke Network (NWORSN) (www.nwostroke.ca). Reflecting on what they had learned about symptoms, risk factors, and the need for early response, elders asked us to extend awareness and prevention messages to children in their communities. They felt strongly that children could serve as messengers, bringing stroke information home to their parents and grandparents.

Health care providers also felt that educating children would improve stroke outcomes: in multigenerational families, where grandparents provide care for children while parents work, children often are the only family member present at the onset of a stroke in an older relative. Health care providers also saw a need to move away from conventional curriculum-based health education products toward creative, flexible, and culturally appropriate tools that could be used in various community settings.

## Community Context

The First Nations communities and indigenous health care organizations who took part in the Teach Our Children study are situated in Northwestern Ontario, Canada. With 231,000 residents scattered over 150,000 square miles, an area equivalent to one-half of the province of Ontario, the region is one of the most sparsely populated parts of North America. (For comparison purposes, it is approximately twice the size of the state of Minnesota, the nearest American neighbor to the south, with less than 4% of that state’s population.) Moreover, this northern region has one of the highest concentrations of indigenous people in Canada: 21.5% of the population self-identify as Ojibwe, Oji-Cree, Cree, or Métis (10). Approximately 10% of the indigenous population lives in Thunder Bay, the region’s urban center; another 40% are in small towns and rural settlements served by year-round roads; the remaining 50% live in 82 remote places reached by air or winter roads, and 31 are air-access only. Most First Nations are small, with 200 to 400 residents; the largest has a population of 2,500 (11).

Because rural and remote First Nations settlements have only nursing staff on site (12), the risk of stroke is compounded by the difficulty of accessing specialized care. People experiencing a stroke in remote places — for example, 275 to 550 miles by air from the regional stroke center — would reach the center in 4 hours, scarcely within the recommended time window for treatment. Adverse weather can cause similar delays when people from remote areas are sent to 4 rural hospitals that provide telestroke care. Given these challenges, teaching children to recognize stroke signs and symptoms and the need for rapid response were priorities in this study.

The major questions addressed in the Teach Our Children project were “What do indigenous youth understand about stroke, what do they need to know about stroke, and how can this information best be delivered to them?” Objectives were to 1) work with children and health care professionals to assess levels of stroke knowledge and preferred educational strategies; 2) engage urban, rural, and remote First Nations communities and indigenous health care organizations in research; and 3) involve indigenous children in developing tools to improve stroke awareness.

## Methods

This community-based project used participatory methods deemed appropriate for research with Canadian indigenous communities (13). The NWORSN Aboriginal Advisory Committee, which includes 19 indigenous health care providers, worked closely with researchers throughout the study, providing guidance on site selection, community engagement, data collection, interpretation of findings, and development of tools. They also helped define a target group, advising that children aged 10 to 13 years (grades 5 through 8) would be most receptive to messages. The study was funded in December 2009 and conducted over a 30-month period, from engagement with communities and health organizations (5 months), through ethics reviews (8 months), to fieldwork (6 months), additional ethics review to facilitate children’s participation in creative product development (3 months), production of the educational tool (4 months), and dissemination to participating communities (4 months). The study was concluded in October 2012.

### Site selection

With guidance from the NWORSN Aboriginal Advisory Committee, the researchers identified regional indigenous communities and health care organizations that could serve as sentinel sites (14). Our goal was to capture issues around geographic access to care and ensure that Ojibwe, Oji-Cree, Cree, and Métis traditions were represented. This study was approved by the Lakehead University Research Ethics Board and the Thunder Bay Regional Health Sciences Centre Research Ethics Board. Once ethics approval was received, invitations were sent to all potential sites. Team members and a local indigenous facilitator traveled to 7 rural First Nations, held 2 video conferences with remote First Nations, and arranged 6 face-to-face meetings with indigenous health care organizations to discuss participation. The 10 First Nations and 4 health care organizations who agreed to support the study differed widely in their geographic isolation, access to specialized services, population served, and traditions (Table 1, Table 2).

**Table 1:** First Nations Communities Participating in Study of Stroke Education for Indigenous Children, Ontario, Canada, 2009–2012

Community	Distance to Regional Stroke Center, Miles	Travel Time, h (by Air)	Population, 2006 (On-Reserve)^a^	Language and Cultural Background
Bingwi Neyaashi Anishinaabek	125	1.25	250	Ojibwe
Fort Severn	535	3.5	500	Cree
Naicatchewenin	270	1.75	260	Ojibwe
Nigigoonsiminikaaning	220	1.5	480	Ojibwe
Pic Mobert	230	1.5	320	Ojibwe
Pic River	200	1.25	480	Ojibwe
Sandy Lake	375	2.5	2,175	Oji-Cree
Seine River	200	1.25	312	Ojibwe
Mitaanjigamiing	250	1.75	100	Ojibwe
Whitesand	160	1.25	350	Ojibwe

a Source: Indigenous and Northern Affairs Canada. First Nation profiles (http://fnp-ppn.aandc-aadnc.gc.ca/fnp/Main/index.aspx?lang=eng).

**Table 2:** Health Care Organizations Participating in Study of Stroke Education for Indigenous Children, Ontario, Canada, 2009–2012

Organizations	Services	Geographic Location, Sites Served	Clients	Cultural Background of Clients
Biidaajiwun Community Health Outreach Centre	Health care and cultural support	Urban	Indigenous families	Ojibwe and Métis
Dilico Anishnawbek Family Care	Health care and cultural support	Rural and urban	Indigenous families	Ojibwe
Keewaytinook Okimakanak Health	Community and regional programs	Remote^a^	Indigenous families	Cree, Ojibwe, Oji-Cree
Thunder Bay Indian Friendship Centre	Health care and cultural support	Urban	Indigenous families	Ojibwe and Métis

a Remote communities are those without year-round road access, which rely on air travel to reach larger communities where hospitals and other specialist services are located.

After permission was obtained from the Chief and Council of each First Nation and executive directors of health organizations, each site identified a health care worker fluent in Ojibwe, Oji-Cree, or Cree to assist. The liaison asked parents and guardians to give approval for children to take part, extended invitations to health care and education professionals to complete interviews, and ensured local traditions, values, and needs were respected.

### Data collection

The research employed a qualitative descriptive design that has proven useful in gathering culturally meaningful data for program planning (15). A common set of questions was developed to assess knowledge of stroke and ideas on how best to deliver messages. Items for discussions with children and provider interviews assessed 1) youth knowledge of stroke signs and symptoms, the need for rapid response, and perceptions of lifestyle risks; 2) opinions on cultural and age-appropriate content that would appeal to young people; and 3) preferences for web-based or DVD media for messaging in diverse settings. 

An indigenous facilitator and nonindigenous researcher traveled to the 10 study sites (7 rural, 2 remote, and 1 urban) to collect qualitative data from children and health care and education professionals. Following traditional protocols and processes (16), we provided honoraria for liaisons, elders, and schools; gifts for participating children; and feasts for children, parents, and elders in all locations.

Overall, 108 children from 10 First Nations and 2 urban indigenous organizations took part; depending on local traditions, talking circles (17) or informal focus groups (18) were used. When talking circles were held, the facilitator gathered children together and presented a talking stick (19), a ceremonial staff used to encourage people to speak from their hearts and respect others. As questions were asked, youth took turns holding the talking stick, taking as much time as needed to speak, before passing it along. When focus groups were employed, the facilitator engaged participants in informal question-and-answer sessions in a comfortable setting, such as a recreation center. A team member observed and documented responses in detailed written notes because audio recording of such discussions was considered disrespectful.

Following data collection, interviews were completed with 26 health care and education professionals who were employed by indigenous organizations serving urban, rural, and remote areas. Notes from participant discussions and verbatim transcription of health care provider interviews were analyzed independently by an indigenous member of the team (R.F.) and 3 nonindigenous researchers (P.B., M.H., G.B.), and coding was compared to identify children’s knowledge needs and make recommendations about preferred ways of delivering stroke messages. Resulting recommendations were consensually validated through a series of discussions between researchers and the NWORSN Aboriginal Advisory Committee.

## Outcomes

### Children’s knowledge about stroke

As expected given their age, indigenous children had limited knowledge about stroke and its signs, symptoms, and consequences. While some thought stroke was a “brain attack” or “brain bleed,” there was confusion about differences between heart attacks and strokes. Those who had a relative who had had a stroke were somewhat more aware of symptoms, citing “one side numbness.” A few knew that high blood pressure or diabetes caused stroke and felt that healthy lifestyles including eating healthfully and being physically active were protective.

However, knowledge varied among participants. Children in remote communities were better informed than those in other locations because of first response training offered as part of the Junior Canadian Rangers (Northern Militia) and the Canadian Red Cross (remote community first aid and injury prevention) programs; these programs were not offered in rural and urban areas. Although children in discussion groups were certain about where they would go for help if they thought someone was having a stroke, their answers reflected resources available locally: they might call the nursing station (smaller rural and remote First Nations), go to a health center or the police (larger rural and remote communities), or dial 911 or 229 if near a hospital with emergency services.

Health care providers emphasized that indigenous children needed to learn more about stroke, because the topic was not taught in schools and likely not discussed at home. Even children who had a relative with stroke would not necessarily realize what was happening. As a nurse said, “a lot of kids will talk about . . . whether grandma or grandpa can’t feel on the right side. . . . Other than that I don’t think they really understand why.” Providers were equally concerned that children would think that stroke was an older person’s illness and would mistake stroke symptoms in a younger person for other problems. Also, a child might not recognize the need for rapid response.

With education, health professionals were confident children would be able to identify signs and symptoms of stroke and know what to do if they thought someone was having a stroke. They believed children could see connections between unhealthy behaviors, such as smoking or taking drugs, and the likelihood of stroke. Also, children could understand that the healthy lifestyles and healthy choices that keep diabetes and blood pressure in check could prevent stroke.

### Preferred educational formats

When asked how they would prefer to hear more about strokes, participants said they wanted to learn in settings other than classrooms. Most liked the interactive DVDs or videos that diabetes educators used to encourage physical activity. They were equally keen about learning via songs and stories. Only a few wanted to learn by using computer or Internet-based games.

Health professionals thought children would learn well through visual formats, such as DVD or Internet presentations. Their views on technology, however, were mixed. Although some felt children were computer savvy and would like web-based learning, this was not feasible in rural or remote places where the Internet was unavailable or unreliable. Others thought children already spent too much time on computers and that additional screen time would be unhealthy.

The consensus among educators was that a DVD was the most effective way to deliver stroke information to children in this age group. Also, they felt messages should be delivered by indigenous children themselves “because kids remember what they see someone else do.” They also recommended storytelling, songs, and music that reflected Ojibwe, Oji-Cree, and Métis traditions. Lastly, they emphasized the need for tools that could be used in varied settings, such as schools, gatherings of children, health fairs, recreational events, or workshops. As a nurse said, “If they’re together for whatever reason, it’s a good time to target them about any kind of health teaching.”

### Development of a creative educational product

The principal outcome from this 3-year community engagement was a creative educational product in the form of a DVD titled Act F-A-S-T 1-2-3! (Video). The video is 11.5 minutes long and features an indigenous elder in the role of a storyteller. Beginning with a visual analogy explaining stroke with images of a river and beaver dam, the elder answered children’s questions by passing a talking stick from one community to another. In each location, children educated their peers about stroke topics (eg, what to do in case of a stroke, risk factors, stroke prevention) through role playing, messaging in the same words used in group interviews, and singing the ACT F-A-S-T Stroke Song that was commissioned to help them remember.

**Video:**
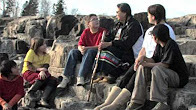


Reflecting our commitment to engagement, the script was reviewed and approved by the NWORSN Aboriginal Advisory Committee. The team also held 2 meetings with 25 elders, who suggested enhancing cultural content by having the children wear the red, white, yellow, and black colors of the indigenous medicine wheel, a symbol of holistic healing, and filming children in natural settings near rivers, forests, and fields that surround their homes.

Once messages were finalized, 2 researchers (P.B. and R.F.) and 2 videographers traveled to 6 rural and 2 remote sites so children, with permission of parents, could participate in the filming. More work was done with children and an elder and researcher (B.M.) from an urban organization. Seventy-five children aged 10 to 13 years contributed directly; as individuals or groups, they enthusiastically delivered positive stroke awareness messages to peers and communities.

At the end of the project, the team returned to the First Nations and indigenous health care organizations that took part in the filming to thank them for their assistance; 157 children, parents, teachers, and community members attended feasts to celebrate. Additional honoraria were provided to the schools and health care organizations who gave direct support.

Following this, the DVD was disseminated to community leaders, health care organizations, and schools serving 70 First Nations and Métis communities across the region. Each DVD included a letter from the chair of the NWORSN Aboriginal Advisory Committee suggesting nurses and educators could use the material for one-on-one teaching and classes for children and adolescents in grades 5 and higher. The letter also encouraged them to show the DVD at health fairs and workshops so parents and elders could enjoy the video and learn along with the children.

## Interpretation

The Teach Our Children project adds to the evidence that the success of indigenous health promotion projects depends to a large extent on the close partnerships formed among researchers, communities, and health care organizations. In this study, we learned that communities are generous in their support when questions meet their needs and studies are done in ways that respect cultural traditions and processes. Our indigenous partners invested considerable time and effort to make sure that the team “got it right.” They were welcoming, helpful, and encouraging in their support of the research and the creation of the DVD.

For those interested in developing similar culturally responsive educational products, practical lessons learned included 1) the need for extensive face-to-face consultation with leadership in potential partner communities and organizations before development to ensure that study goals, objectives and methods were acceptable; 2) the value of working closely with liaison persons to ensure that research reflects community needs, preferences, and priorities and follows local traditions; 3) the recognition that extra time is required for ethics reviews to accommodate differences between standard academic guidelines, indigenous community expectations, and local protocols; and 4) the need to advocate strongly for sufficient funding to support engagement, fieldwork, and dissemination in indigenous communities, because the costs of traveling to geographically isolated areas to do the work are significant.

Although this study underscores the benefits of collaborative partnerships, we recognized that the project, in and of itself, represented only one step in the process of making sure that stroke messages for indigenous children meet identified needs. Limited resources and the time frame of funders did not permit a comprehensive follow-up to assess the generalizability of the DVD to indigenous communities with different cultural beliefs. The study also could not answer questions about whether this product, developed for children living in northern urban, rural, and remote areas, would be effective with indigenous children who live in large metropolitan centers who may identify more strongly with global youth culture than with their own traditions (20).

Two years after project completion, the NWORSN Aboriginal Advisory Committee asked us to conduct a brief telephone survey of selected rural and remote First Nations to assess how the video was used. Results indicated that the DVD was used to teach children in schools, health centers, and community settings in 12 of the 20 sites surveyed. Informal discussions with health educators also confirmed that Teach Our Children had broad appeal and was used to instruct indigenous adults and elders in stroke and diabetes prevention programs across the province.

The study demonstrates that effective engagement can lead to culturally and age-appropriate health promotion products. The DVD, blending storytelling traditions, songs, and messages delivered by indigenous children to their peers, attests to the value of collaboration. It adds to the evidence that working closely with indigenous communities and organizations is a critical component of developing successful educational strategies for youth and their families.
